# Design, fabrication and characterization of metamaterial absorber for sensing applications

**DOI:** 10.1038/s41598-026-37524-9

**Published:** 2026-03-04

**Authors:** Doaa Mohammed Marie Helaly, Mohamed Farhat O. Hameed, Nihal F. F. Areed, S. S. A. Obayya, Ahmed S. Saadeldin

**Affiliations:** 1 Electronics and Communications Department, Faculty of Engineering, University of Mansoura, Mansoura, 35516 Egypt; 2 Centre for Nanotechnology, Zewail City of Science, Technology and Innovation, October Gardens, 6th of October City, Giza 12578 Egypt; 3 Department of Mathematics and Engineering Physics, Faculty of Engineering, University of Mansoura, Mansoura, 35516 Egypt; 4 Department of Electrical Engineering, College of Engineering, Qassim University, 52571 Buraydah, Saudi Arabia; 5 Centre for Photonics and Smart Materials, Zewail City of Science, Technology and Innovation, October Gardens, 6th of October City, Giza 12578 Egypt; 6 Electronics and Electrical Communications Department, Akhbar Elyom Academy, 6th of October, Giza Egypt

**Keywords:** Biomedical sensor, Perfect absorber, Refractive index sensing, Metamaterial sensor, Engineering, Materials science, Optics and photonics, Physics

## Abstract

A perfect metamaterial absorber (MMA) is suggested, analyzed, and fabricated for sensing applications. The reported design enhances the absorption at the resonance frequency by suppressing the reflectance and transmittance concurrently. A back metallic reflector is used to eliminate the transmission through the proposed design, while the reflection is minimized by the impedance matching between the proposed MMA and that of free space. Moreover, simultaneous electric and magnetic resonances are supported with nearly perfect absorption at the resonance frequency. The reported design has simulated absorptivity of 99.33% at 28.146 GHz which falls within the mm-Wave spectrum, a band of critical importance for next-generation 5G communication and high-frequency biosensing. Further, the fabricated absorber achieves a measured absorption of 96.5% at 28.12 GHz with a narrow absorption bandwidth. In addition, the resonance frequency strongly depends on the surrounding refractive index, which makes the absorber an effective platform for RI sensing. The simulated sensitivity of the proposed RI sensor is 9520 GHz/RIU when the analyte refractive index changes from 1.3 to 1.35 with simulated Q-factor and FoM of 817 and 427 RIU^-1^, respectively. A prototype is fabricated and characterized with excellent agreement with the simulation results. High measured sensitivity of 14.44 GHz/RIU is obtained (the analyte layer changed from air to water) from the prototype characterization with a Q-factor of 513 and FoM of 427 RIU^-1^. The potential of using the proposed metamaterial absorber as a biosensor for cancer cell detection is also demonstrated for Breast cell, Cervical cell, Jurkat cell, MCF-7 cell, and PC12 cell with an average simulated sensitivity of 9.023 GHz/RIU. Therefore, the reported perfect metamaterial absorber has good potential in sensing applications.

## Introduction

Metamaterials (MMs) are materials with properties not found in nature. These properties are engineered by manipulating materials at scales smaller than the wavelength of electromagnetic waves. This results in unique features like a negative refractive index^1^, near-perfect absorption^2^, anomalous dispersion^3^, and tunability^4^. These extraordinary properties enable groundbreaking applications^5^ such as subwavelength imaging^6^, superlenses^7^, invisibility cloaking^8^, waveguiding, and inverse Doppler Effect^9^, lasing^10^, and asymmetric light transmission^11^. Additionally, many applications have been demonstrated based on perfect absorption of electromagnetic (EM) energy, such as solar cells^8^, photonic/thermal imaging^12^, sensors^13,14^, radiative cooling^15^, narrowband selective filters^16^, and thermal emitters^17–19^. Therefore, researchers were making remarkable progress in developing efficient metamaterial absorbers with sharp ^20–22^ or wide absorption bands^23–25^. Additionally, single-band^20^ or multi-band metamaterial absorbers^26^ can be achieved successfully. In 2008, Landy proposed the first realized metamaterial absorber (MMA) with an absorption peak of 88% at 11.5 GHz by using an electric ring resonator (ERR)^8^. The MMA can be used in sensing applications due to the remarkably achieved narrow-band behavior. In 2022, Islam et al.^27^ designed a sensor with a reflection coefficient of − 23.87 dB at 9.46 GHz for the recognition of various oils, fluids, and chemicals with a unit cell dimension of 22.86 × 10.16 mm. Such a sensor has a sensitivity of 0.56% with a quality factor of 135. The same team proposed a better design with a reflection coefficient of 56.71 dB at 10.21 GHz^28^ based on three square resonators. The new design has a better sensitivity of 1.99% with a higher quality factor of 430 than that reported in^27^. Further, split-ring resonator-based MMA has been suggested^29^ with a rectangular-shaped reflected mirror with a reflection coefficient of −14.99 dB at 9.06 GHz and a reflection coefficient of −9.35 dB at 11.16 GHz. The unit cell of the proposed design has a dimension of 22.86 × 10.16 mm that can be used for X-band applications. The sensitivity, Quality (Q)-factor, and Figure of Merit (FoM) were equal to 1.29%, 435, and 561.15, respectively, around a frequency of 9.06 GHz. However, the corresponding values were equal to 1.13%, 325, and 367.25 around a frequency of 11.16 GHz. Furthermore, Khalil et al.^30^ presented a double negative chiral quadruple square resonator-based MM sensor with a unit cell dimension of 20 × 20 mm that is suitable for the S-band applications. The proposed sensor has two resonant frequencies, one at 2.88 GHz with a reflection coefficient of − 44.84 dB, while the other peak occurs at 3.57 GHz with a reflection coefficient of − 19.8 dB. The sensitivity, Q-factor, and FoM are equal to 0.661%, 1413.29, and 934.184, respectively, for the lower resonance frequency. However, the corresponding values for the upper peak are equal to 0.19%, 1140.16, and 21.975, respectively. In ^31^, Liang et al. proposed a dual-band MM sensor with a high Q-factor of 665.85 and 1883.3 at 5.46 GHz and 11.3 GHz with reflection coefficients of -31 dB and -38 dB, respectively. Such a design consists of a cross metallic structure and a ring resonator on a 15 × 15 mm substrate for refractive index sensing applications. In this context, sensitivities of 0.85 GHz/RIU and 1.48 GHz/RIU were achieved at 5.46 GHz and 1.3 GHz, respectively. Further, Al Zafir et al.^32^ presented an epsilon-shaped metamaterial absorber with a dimension of 10 × 10 mm, for K-band and Ka-band applications operating at resonance frequencies 20.193 GHz and 30.087 GHz with an absorbance of 99.5% and 99.9%, respectively. The quality factors for both resonances are equal to 155.33 and 527.84, respectively. In^33^, Cao et al. introduced a 13 × 13 asymmetric electric split-ring resonator (AESRR) MM structure where a new Fano resonant peak was obtained at *f* = 11.575 GHz with a reflection coefficient of − 13.2 dB and a sensitivity of 0.612% for sensing the dielectric environmental change. Recently, dual-band and multiband metamaterial absorbers have been studied extensively. In^34^, V-shaped metamaterial absorber has been reported to achieve dual-band perfect absorptivity of 99.54% and 99.98% at 10.89 GHz and 16.33 GHz, respectively. Such a design is introduced as a pressure sensor with a sensitivity of 2.9 MHz/ µm. Further, Moniruzzaman et al. have presented a dual-band metamaterial absorber at 1.8 GHz and 3.5 GHz with absorptivity of 98.7% and 99.7%, respectively^35^. Additionally, Penta band metamaterial absorbers have been introduced^36^ and^37^ with absorptivity above 90% in Gigahertz and Terahertz ranges, respectively. Furthermore, an Archimedes-based metamaterial absorber is proposed for refractive index sensing applications in the X-band range^38^. This design has seven absorption bands with absorptivity between 77.1 and 99.9%. In^39^, a compact quadrilateral half-circle resonator metasurface for microwave sensing applications has been presented. A dual absorption has been achieved at 21.45 GHz and 23.04 GHz with absorptivity above 90%. This design has been used to detect changes in the dielectric constant of the analyte layer with sensitivity up to 1.1 GHz/unit.

The MM sensor can be used for cancer early detection^40–43^, which has an important health, social, and economic impact due to the increase in the number of new cancer cases and deaths^44,45^. The MM sensor offers many advantages over the conventional techniques, such as electrochemical methods^46^, histopathological or immunological methods^47^, computed tomography^48^, fluorescence imaging^49^, and cytometry^50^ These techniques suffer from restrictions such as imprecision, high costs, complexity, long duration, radiation exposure and the requirement for trained staff^51–53^. While the MM sensor offers solutions and privileges to these limitations, it comes with better enhanced sensitivity and resolution, tunable properties to many biomarkers, lightweight and miniaturized designs, real-time detection, non-invasive technique, effective cost, multiplexing capability to detect multiple biomarkers simultaneously, specificity, and integration with other technologies^53–55^. In 2025, Hamza et al. designed an MM-based triple-band biosensor for early-stage cervical cancer detection with a sensitivity of 0.049 THz/RIU, Q-factor of 85.77, and a FoM of 6.3 ${\mathrm{RIU}}^{-1}$ at 0.68 THz for the first resonance frequency^56^. While for the third resonance frequency, the proposed design provides a sensitivity of 0.068 THz/RIU, Q-factor of 41.46, and a FoM of 3 ${\mathrm{RIU}}^{-1}$ at 0.971 THz. However, the second resonance has been neglected as it didn’t reach the perfect absorption (80%). In 2025, Aliouar et al. designed a MM biosensor within the terahertz frequency spectrum to identify cutaneous malignancies^57^. This design gives maximum absorption efficiency of 98.84% at a frequency of 3.257 THz, accompanied by a remarkable Q-factor of 325.7 with an average sensitivity of 897 GHz/RIU, and a FoM of 89.7 RIU⁻1. In 2024, Rezeg et al. proposed a highly sensitive graphene-based metamaterial biosensor for early cancer detection in the THz frequency band by employing three circular graphene split ring resonators^58^. It has a resonance frequency at 5.662 THz, with a remarkable sensitivity of 3.880 THz/RIU, and a 8.14 ${\mathrm{RIU}}^{-1}$ FoM and a high-quality factor of 8.948. In 2024, Hamza et al. represented a three-band THz Metamaterial sensor that offers a promising avenue for the fast, highly accurate, and non-invasive diagnosis of blood cancer^59^. The first peak at 0.4728 THz with absorption of 99% has The minimum sensitivity, 930 GHz/RIU and a Q-factor of 24.86 and a FoM of 42.6 ${\mathrm{RIU}}^{-1}$, the second peak exist at 0.69826 THz with absorption of 90.5% has a sensitivity of 1630 GHz/RIU and a Q-factor of 24.83and a FoM of 50.6 ${\mathrm{RIU}}^{-1},$ the third peak exist at 0.7901 THz with absorption of 92% has the maximum sensitivity that equal 2540 GHz/RIU and a Q-factor of 34.74 and a FoM of 98.6 ${\mathrm{RIU}}^{-1}$. In 2024, Etman et al. proposed a highly sensitive metamaterial (MM) sensor fabricated for biomedical applications based on a circular copper layer etched with four squares over an FR-4 dielectric substrate^60^. It has a high absorption peak of 99.9% at 36.508 GHz resonance frequency with a high-quality factor of 23.1. Additionally, a high average sensitivity of 5.7 GHz/RIU. In 2023, Dadouche et al. suggested an original experimental biosensor for cancer cell detection using a corona-shaped metamaterial resonator^61^. It has an absorption peak of 94.1% at 2.988 GHz resonance frequency. This resonator is designed to detect cancer markers with a high sensitivity of 0.1825 GHz/RIU. These properties permit the biosensor to effectively differentiate many different types of cancer cells, including basal cell, breast and cervical, Jurkat, MCF-7, and PC12. In 2023, Elhelw et al. suggested a highly sensitive triple-band metamaterial-based biosensor operating in the THz frequency band; only the third peak at 4.101 THz is considered in the stuof 55more sensitive to the variation in the refractive index of the analyte samples as it has a sensitivity of 2050 GHz/RIU, a FoM of 25.24 ${\mathrm{RIU}}^{-1}$, and a Q-factor of 55.34^41^. In 2022, Singh et al. proposed an ultrathin metamaterial absorber for the refractive index detection of biomedical samples^62^. The absorber is made up of a novel spanner resonator and has an abortion peak of 99.7% at 105.7 GHz with a Q factor of 19.57, an average sensitivity of 14.81 GHz/RIU, and a FoM of 3.48.

In this paper, a metamaterial absorber exhibiting near-ideal performance is investigated through comprehensive numerical simulations and experimental validation. The proposed structure is composed of a compact unit cell with an overall footprint of 15 mm × 15 mm (with unit cell size in terms of resonance wavelength of 1.4 λ × 1.4 λ × 0.075 λ). The electromagnetic response is rigorously analyzed using a three-dimensional full-wave solver implemented in the commercial CST Microwave Studio platform^63^. Then, a prototype of a perfect metamaterial absorber (10-unit cells × 10-unit cells) is fabricated, characterized, and compared to the simulated results. The metamaterial absorber has a measured absorptivity of 96.5% at 28.12 GHz with a Q factor of 513. The high absorption coefficient is due to the strong field confinement around the upper metallic layers with electric and magnetic resonances. Additionally, negligible cross-polarization (S_yx_) is obtained. The resonance frequency depends on the surrounding medium RI with a high Q-factor, which can be used for sensing applications. In this study, the resonance frequency is shifted to 23.5 GHz (when the analyte layer changed from air to water) with an absorptivity of 95% and sensor sensitivity of 14.44 GHz/RIU. The potential of using the reported MM as a biomedical sensor for cancer early detection is also demonstrated. The proposed biosensor achieves an average sensitivity of 9.023 GHz/RIU for sensing different types of cancer cells, such as Basal, Breast, Cervical, Jurkat, MCF7, and PC12 cells, which is proof of its effectiveness as a biomedical sensor. It is worth noting that the proposed design, as a cancer cell sensor, demonstrates superior performance compared to previous designs in the Gigahertz range with a simple design. By comparing our results with those reported in^60–62^, the proposed design exhibits higher normalized sensitivity, higher Q-factor, and higher FoM, with values of 51.62% RIU^-1^, 817, and 427 RIU^-1^, respectively. Furthermore, the excellent agreement between the simulated results and the experimentally characterized prototype demonstrates the design’s robustness and its practical feasibility for high-precision biosensing.

## Design considerations

**Fig. 1:**
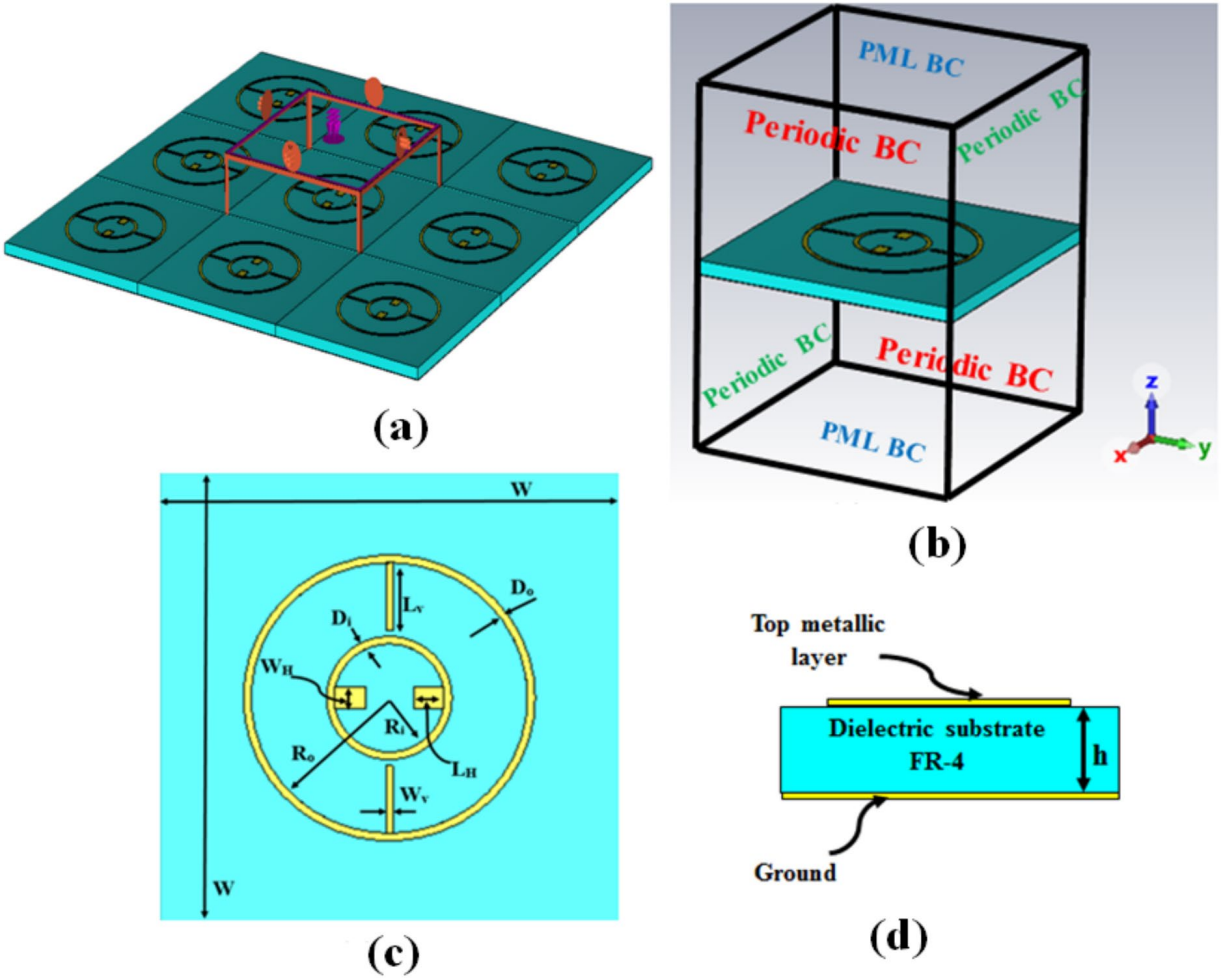
Schematic diagram of the proposed metamaterial absorber; (**a**) 3D, (**b**) unit cell, (**c**) top view, and (**d**) side view.

Figure 1a and b show the 3D and unit cell of the proposed design, while the top view and side view are shown in Fig. 1c and d, respectively. The suggested perfect MMA is composed of three layers, two copper layers separated by a 0.8 mm-thick FR-4 epoxy dielectric substrate (${\varepsilon }_{r}$ = 4.6, loss tan $\delta$ = 0.019). The FR-4 material is used due to its low cost, minimum loss, and superior mechanical properties. The bottom layer is a layer of copper with a thickness of 0.035 mm, which acts as a perfect reflector. The top layer consists of two concentric circular copper rings with a thickness of 0.035 mm and a conductivity of 5.96 $\times {10}^{7}$ S/m^33^. The initial geometrical parameters are summarized in Table 1 and are inspired by the literature^64–66^ to facilitate the fabrication of the reported design. The performance of the proposed absorber was initially examined using CST Microwave Studio 2021, using the frequency-domain solver. The applied boundary conditions are taken as a perfectly matched layer (PML) along the z-axis for the incident EM wave, while periodic boundary conditions (PBC) along the x- and y-axis as shown in Fig. 1b. In this study, maximum element size in free space of λ_res_/20 is used with a minimum element quality of 0.00808917 and a total number of elements of 58,709 tetrahedrons. Further, the minimum edge length is 0.00173645.

**Table 1:** Initial geometrical parameters of the reported metamaterial sensor.

Geometrical Parameter	Values
$w$	15 mm
${R}_{i}$	1.8 mm
${D}_{i}$	0.25 mm
${L}_{h}$	0.8 mm
${W}_{h}$	0.25 mm
${R}_{o}$	4 mm
${D}_{o}$	0.25 mm
${L}_{v}$	1.25 mm
${W}_{v}$	0.25 mm

The absorption through the proposed design can be obtained by:

1 $$A\left(\omega \right)=1-T\left(\omega \right)-R\left(\omega \right)$$

where $T\left(\omega \right)$ and $R\left(\omega \right)$ denote the transmittance and reflectance, respectively. A perfect absorber can be realized by suppressing the reflectance and transmittance. When the thickness of the bottom continuous metallic layer greatly exceeds the skin depth of the electromagnetic waves, transmission is effectively suppressed, leading to the following expression for absorption:

2 $$A\left(\omega \right)=1-R\left(\omega \right)$$

The scattering S-parameters are commonly used to evaluate the effective permittivity (${\varepsilon }_{eff}$) and effective permeability (${\mu }_{eff}$) of the MMA^50^ as follows:

3 $${\varepsilon }_{eff}=1+\frac{2j}{{K}_{0}h}\left(\frac{1-{S}_{11}}{1+{S}_{11}}\right)$$

4 $${\mu }_{eff}=1+\frac{2j}{{K}_{0}h}\left(\frac{1+{S}_{11}}{1-{S}_{11}}\right)$$

where ${K}_{0}$ is the free space wavenumber, *h* is the substrate thickness, and ${S}_{11}$ is the reflection coefficient. The relative impedance (Z_r_) is defined as the ratio of the metamaterial absorber impedance to free space impedance, which can be written as:

5 $${z}_{r}=\frac{\mathrm{Z}}{{Z}_{o}}=\sqrt{\frac{{\upmu }_{eff}}{{\varepsilon }_{eff}}}$$

**Fig. 2:**
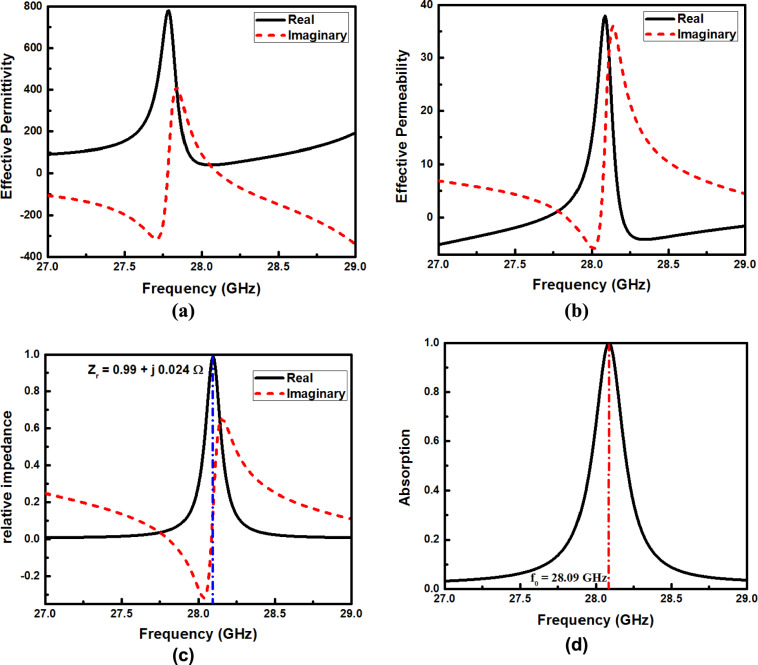
Variation of the (**a**) effective permittivity, (**b**) effective permeability, (**c**) relative impedance, and (**d**) the absorption of the suggested MMA with the frequency.

The geometrical parameters are optimized to facilitate the impedance matching with free space. At the resonance frequency, the relative impedance (Z_r_) approaches unity, where the characteristic impedance of the metamaterial matches that of free space (Z = Z_0_). Therefore, the reflection will be minimized while the absorption will be maximized. The relative impedance Z_r_ of the proposed design can also be extracted from the S-parameters using Eq. (6) ^50^

6 $${z}_{r}=\sqrt{\frac{{\left(1+{S}_{11}\right)}^{2}-{S}_{21}^{2}}{{\left(1-{S}_{11}\right)}^{2}-{S}_{21}^{2}}}=\frac{1+{S}_{11}}{1-{S}_{11}}$$

where the transmission coefficient ${S}_{21}=0$ due to the back reflector metallic layer. Figure 2a and b depict the frequency-dependent variations of the real and imaginary components of the effective permittivity and permeability, respectively. Furthermore, Fig. 2c illustrates the corresponding real and imaginary parts of the relative impedance. As observed in Fig. 2a and b, the effective permittivity attains a value of 41.48 + j8.92, while the effective permeability is 39.96 + j10.51. Consequently, the ratio $\sqrt{{\mu }_{eff}/{\varepsilon }_{eff}}$ is nearly equal to unity at the resonance frequency. Therefore, the absorption behavior arises from the concurrent excitation of electric and magnetic resonances. Figure 2c presents the real and imaginary components of the impedance of the proposed perfect MMA, normalized with respect to free space impedance (i.e., relative impedance). As shown in Fig. 2c, the real part approaches unity while the imaginary part approaches zero, indicating that the impedance of MMA is well-matched to free space impedance, Z_0_ = 377 + j0 Ω. This impedance matching minimizes the reflectance from the proposed structure.

In this study, the proposed MMA can be used as a refractive index (RI) sensor, and its performance and efficiency are evaluated and characterized based on three parameters: Sensitivity, FoM, and Q-factor. The sensitivity measures how much the sensor’s output changes in response to variations in the studied parameter^67^, and is given by:

7 $$S=\frac{{\Delta f}_{0}}{\Delta n}$$

where ${\Delta f}_{0}$ denotes the shift in the resonance frequency, and $\Delta n$ represents the variation in the refractive index of the tested medium.

The FoM represents the sensor’s overall capability to distinguish between different signals^30^*,* and can be calculated from the following equation:

8 $$FoM=\frac{S}{FWHM}$$

where *S* is the sensor sensitivity and $FWHM$ is the full width at half maximum. The Q-factor is a key parameter that characterizes the sharpness and stability of the resonance, reflecting the sensor’s ability to sustain a strong and consistent response against temporal or frequency fluctuations^30^, and is calculated by:

9 $$Q factor = \frac{{f}_{0}}{FWHM}$$

where ${f}_{0}$ is the resonance frequency. Figure 2d illustrates the simulated absorption of the suggested absorber under normal incidence for transverse electric (TE) polarization. The geometrical parameters shown in Table 1 are employed in this analysis. As depicted in Fig. 2d, the absorber achieves an almost perfect simulated absorptivity of 99.33% at 28.09 GHz under normal incidence with an FWHM of 0.034 GHz. Therefore, strong frequency selectivity is achieved owing to its narrow absorption bandwidth with a high Q-factor of 817 that can be used for sensing applications.

## Numerical results

**Fig. 3:**
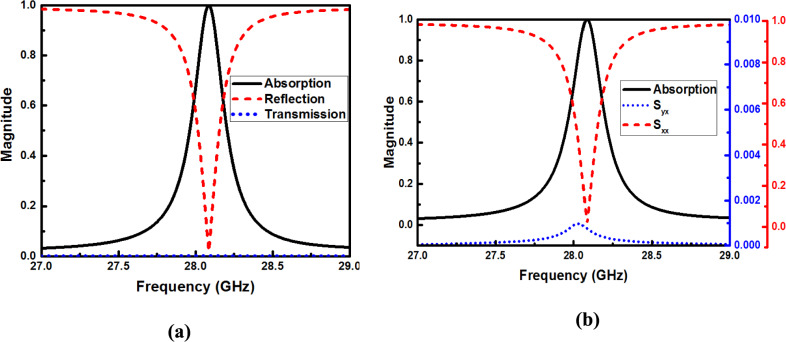
(**a**) The absorption, reflection, and transmission of the proposed MMA, and (**b**) absorption with co-polarized and cross-polarized reflection coefficient.

The absorption shown in Eq. 2 can be expressed in terms of the S- parameters as $A\left(\omega \right)=1-{S}_{xx}-{S}_{yx}$ ^35^ to explicitly account for both co-polarized (${S}_{xx}$) and cross-polarized (${S}_{yx}$) reflectance components. Here, ${S}_{xx}={\left|{s}_{xx}\right|}^{2}$ and ${S}_{yx}={\left|{s}_{yx}\right|}^{2}$ where ${s}_{xx}$ and ${s}_{yx}$ represent the co-polarized and cross-polarized reflection coefficients, respectively. Figure 3a illustrates the absorption, reflection, and transmission characteristics derived from the S-parameters of the proposed design. A near-perfect absorption of 99.33% is achieved at 28.09 GHz with zero transmission. This is due to the metallic ground plane and the minimization of reflection through optimized impedance matching. Figure 3b illustrates the absorption characteristics derived from these specific parameters. As evidenced by the plots, since the cross-polarized reflection coefficient ${s}_{yx}$ remains negligible across the operating band, the resulting absorption spectrum aligns perfectly with that of a conventional incident wave, confirming the polarization purity of the proposed structure.

**Fig. 4:**
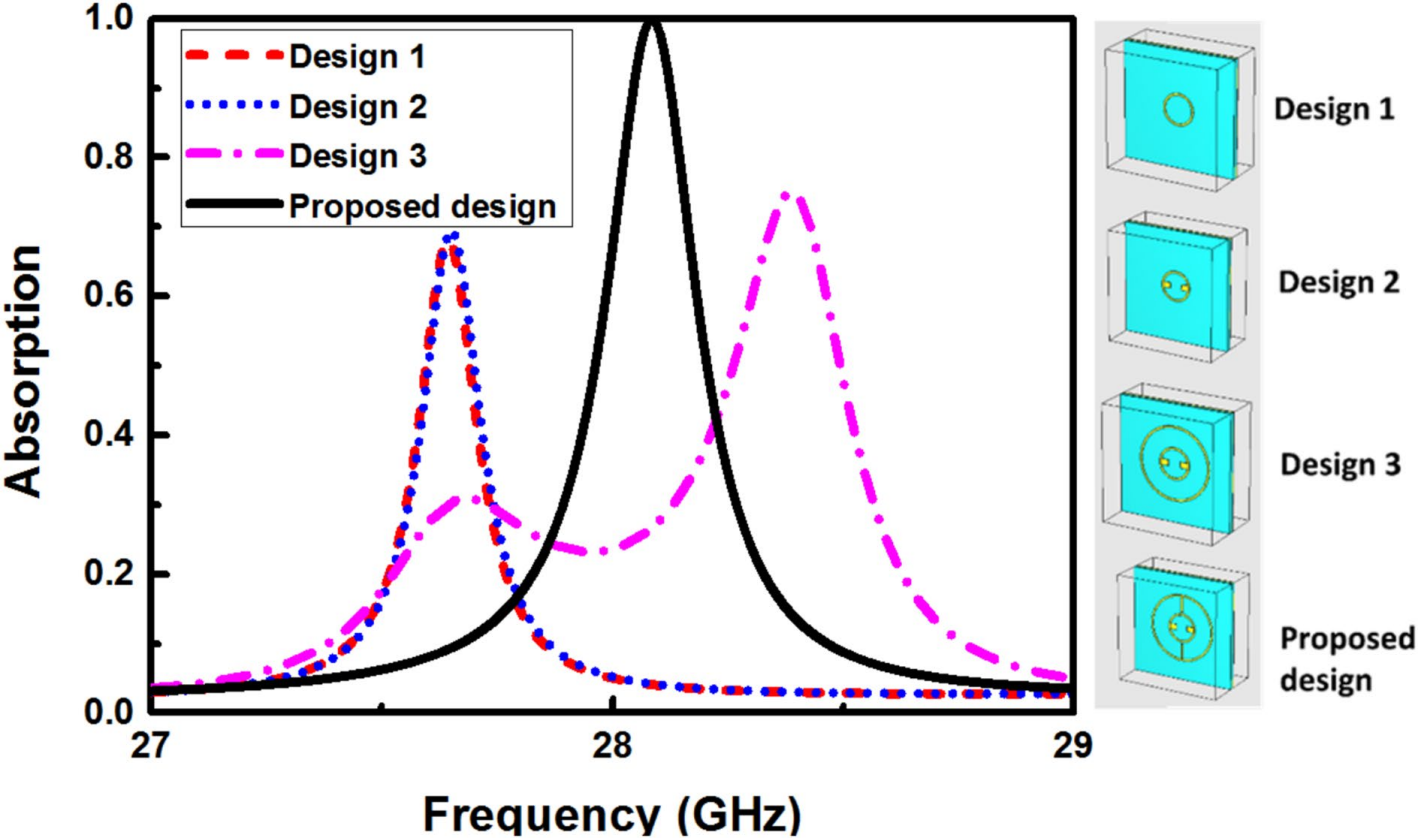
Absorption response of the studied four MMA designs.

To highlight the contribution of each component in the proposed design, the structures depicted in the inset of Fig. 4 were analyzed. Initially, a metamaterial absorber featuring only the metallic ring was tested, yielding an absorption peak at 27.658 GHz with absorptivity values of 65%. Subsequently, two horizontal rods were introduced into the metallic ring (design 2), supporting one resonance peak at 27.7 GHz, achieving an absorptivity of 68%. Next, design 3 was examined, resulting in two resonance peaks at 27.71 GHz, and 28.37 GHz with absorptivity levels of 30.9%, and 74.56%, respectively. Finally, the proposed design was examined, delivering an absorption peak across the frequency 28.09 GHz, with absorptivity exceeding 99.33%.

**Fig. 5:**
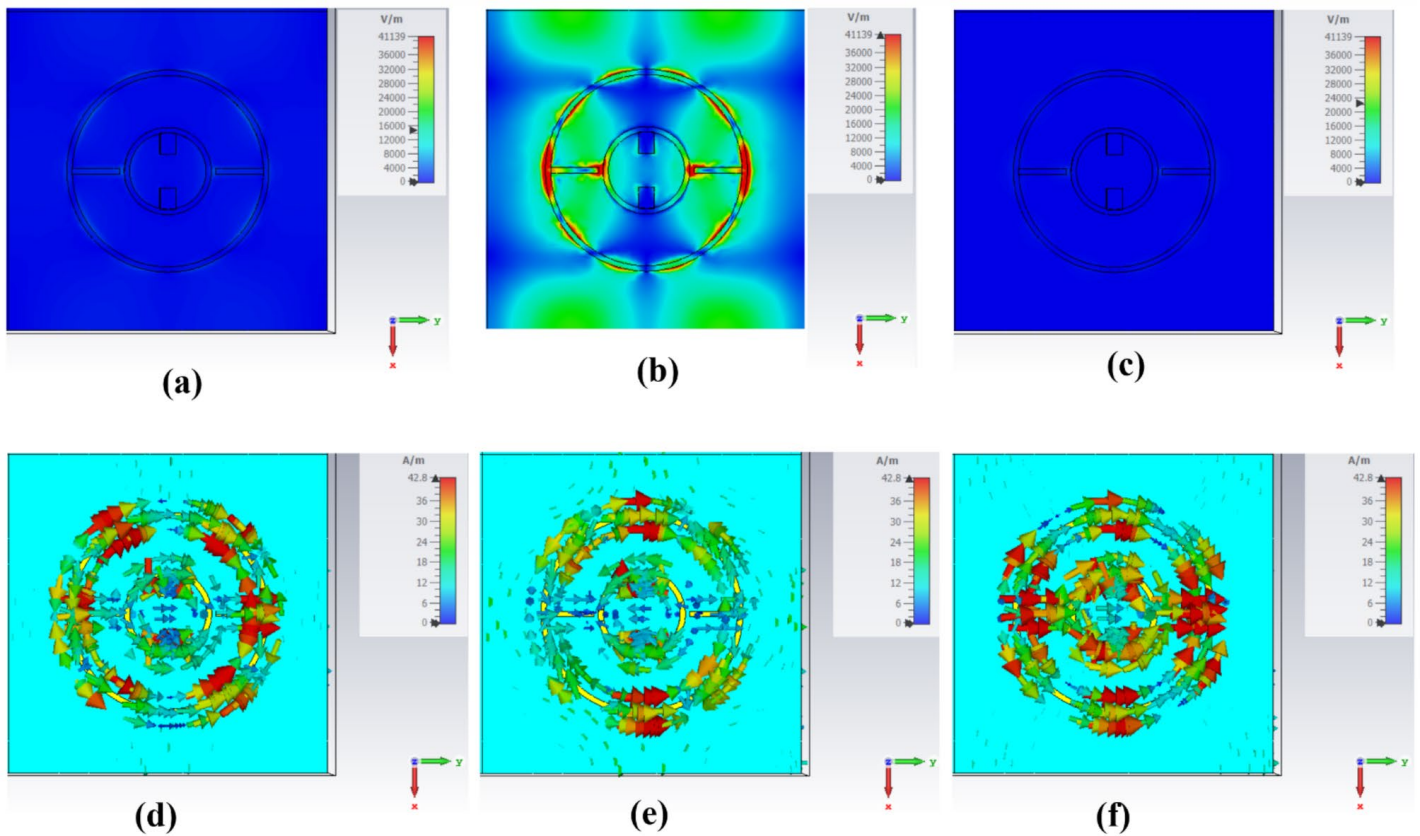
the norm component of the electric field distribution at operating frequencies of (**a**) 26 GHz, (**b**) 28.09 GHz, and at (**c**) 30 GHz. Norm components of the surface current distribution are shown at frequencies of (**d**) 26 GHz, (**e**) 28.09 GHz, and (**f**) 30 GHz.

The electric field distributions at different frequencies (26 GHz, 28.09 GHz, and 30 GHz) are obtained and presented in Fig. 5a, b, and c to understand the absorption behavior of the proposed design. However, Fig. 5d, e, and f show the corresponding surface current at the same frequencies. As observed from Fig. 5, there is a strong confinement of both the electric field and the surface current at the resonance frequency of 28.09 GHz. Therefore, the proposed absorber can effectively absorb the incident wave and is highly sensitive to small variations in the surrounding refractive index, making it valuable for sensing applications. Additionally, a circulating current is induced around the top copper layer^41^ under the excitation of the incident EM wave. Further, the split gaps in the top copper layer introduce capacitance, resulting in an inductive–capacitive (LC) resonance, where the resonance frequency is inversely proportional to $\sqrt{LC}$.

**Fig. 6:**
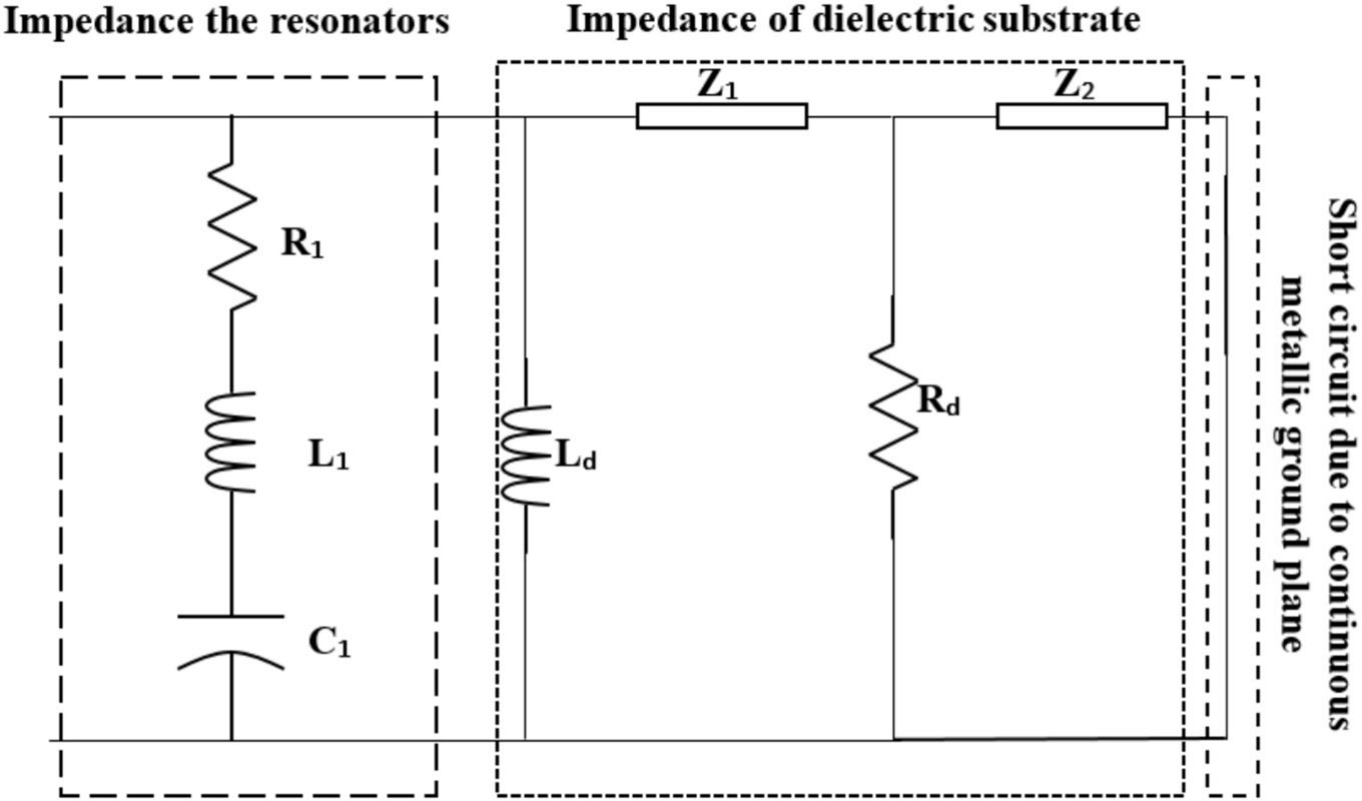
The circuit model for the proposed MMA unit cell.

To provide clearer insight into the operating principle of the proposed MMA, we employed an equivalent circuit model, a methodology previously validated in the works of Nguyen et al.^69^ and Saadeldin et al.^70^. Nguyen et al. achieved over 90% absorption across the 8.2–13.4 GHz range by using three parallel RLC branches to represent specific resonance peaks, while Saadeldin et al. reached similar absorptivity across the 12–20 GHz band with distinct peaks at 13, 17.1, and 19.9 GHz. In this context, Saadeldin et al. utilized three series RLC branches connected in parallel to model their reported design. Because our structure shares a similar underlying physics but with only one peak, the top metallic layer is represented by a single series RLC branch. In this model, L_d_, R_d_, Z_1_ and Z_2_ represent the dielectric substrate as shown in Fig. 6. Additionally, the continuous metallic ground plane is described as a short circuit due to its perfect reflectivity.

**Fig. 7:**
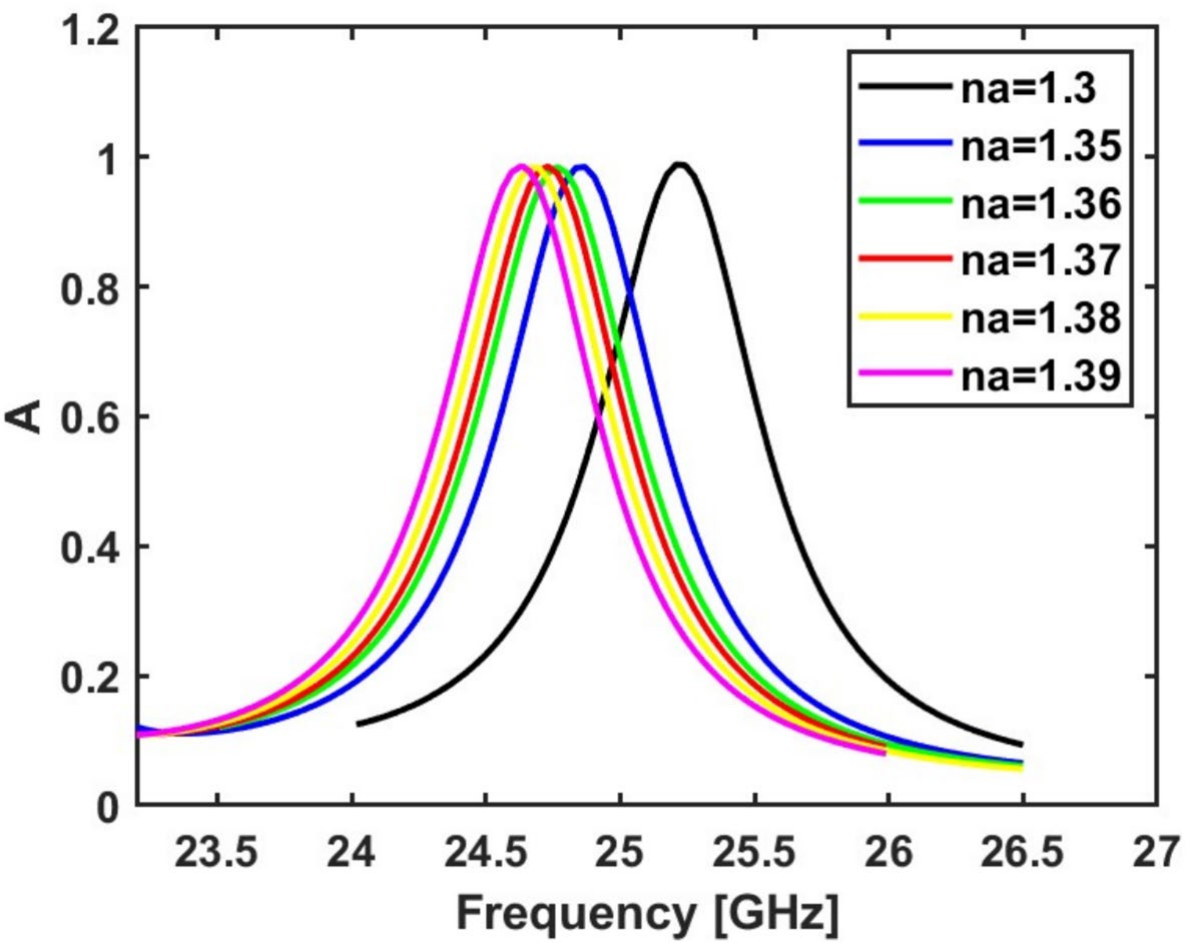
The absorption spectrum of the suggested MMA at different analyte refractive indices. During this study, the geometrical parameters have the initial values (${R}_{i}$ = 1.8 mm, ${D}_{i}$ = 0.25 mm, ${L}_{h}$ = 0.8 mm, ${W}_{h}$ = 0.25 mm, ${R}_{o}$ = 4 mm, ${D}_{o}$ = 0.25 mm, ${L}_{v}$ = 1.25 mm, and ${W}_{v}$ = 0.25 mm.)

The performance of the proposed design as an (RI) sensor is subsequently examined by placing an analyte layer on the defective top copper surface with analyte refractive index (n_a_). A convergence study is conducted on the analyte layer thickness to evaluate its influence on sensor sensitivity. The results indicate convergence at t = 2.11 mm, which is therefore adopted in the subsequent simulations. Figure 7 presents the absorption response as a function of the frequency for various analyte refractive indices. As observed, the resonance frequency shifts with changes in the RI of the analyte layer. The geometrical parameters are then studied to maximize the sensor sensitivity when the analyte RI (n_a_) changes from 1.3 to 1.35. During this study, the accepted minimum absorption is 80%. To interpret well the reason behind the enhancement in sensitivity in each step, we will use the equivalent circuit model as given by^41^. The resonance frequency of the MMA is:

10 $${f}_{o}=\frac{1}{2\pi \sqrt{{L}_{eq}{C}_{eq}}}$$

where *f*_*o*_ is the resonance frequency, ${L}_{eq}$, and ${C}_{eq}$ are the equivalent inductance and capacitance of the sensor, which depend basically on the geometric parameters such as unit cell size, resonator shape and dimensions, and substrate thickness and material properties of the sensor, such as substrate dielectric constant and the conductivity of the metal layers. The inductive elements ${L}_{eq}$ arise from current loops in the metallic patterns, the capacitive elements ${C}_{eq}$ result from the space between metallic patches and the ground plane, and the resistive elements *R* represent losses and are important to achieve impedance matching with free space.

**Fig. 8:**
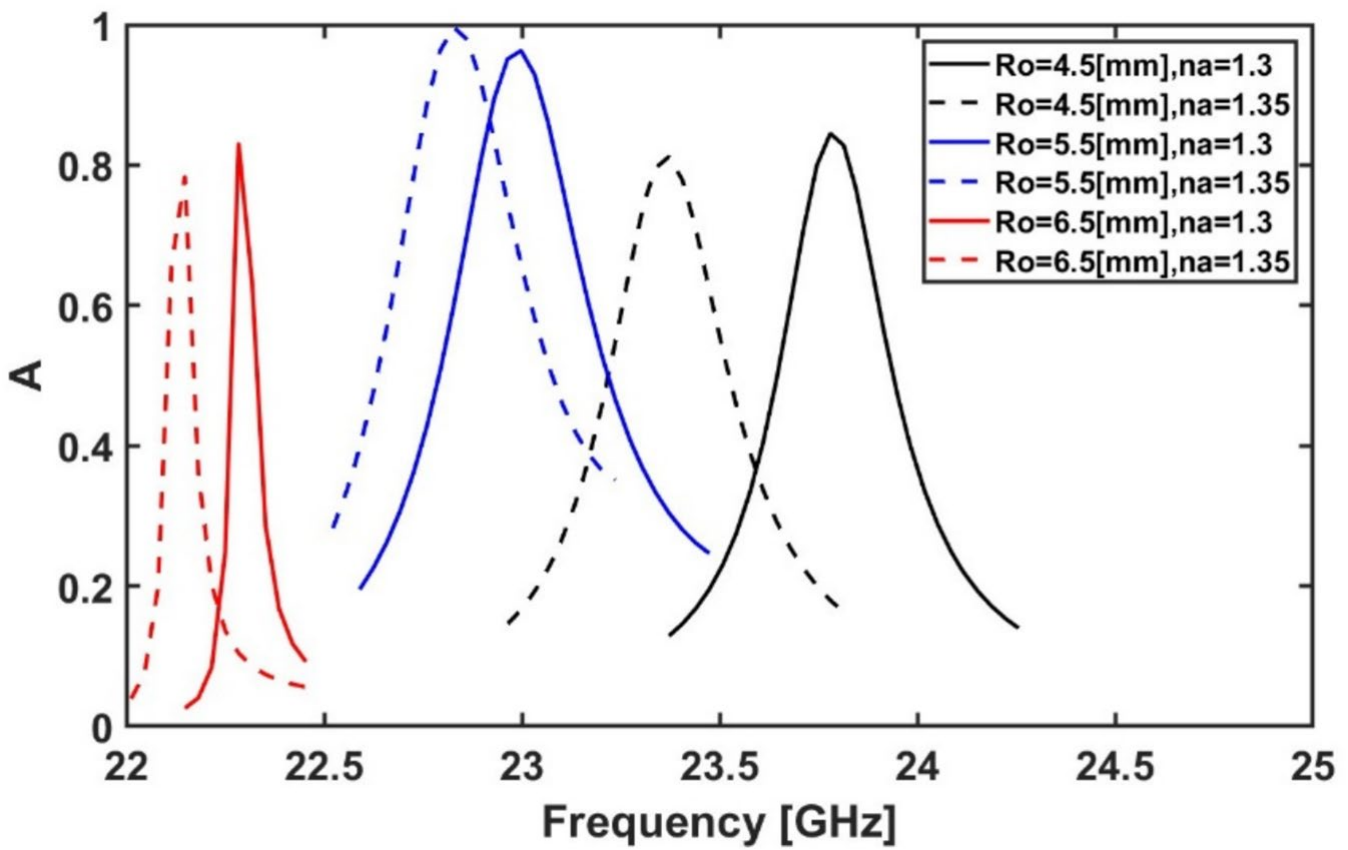
Variation of the absorption spectrum at different radii of the outer circle Ro while the other geometrical parameters remain constant at their initial values (${R}_{i}$ = 1.8 mm, ${D}_{i}$ = 0.25 mm, ${L}_{h}$ = 0.8 mm, ${W}_{h}$ = 0.25 mm, ${D}_{o}$ = 0.25 mm, ${L}_{v}$ = 1.25 mm, and ${W}_{v}$ = 0.25 mm.)

First, the effect of ${R}_{o}$ on the sensor sensitivity is studied by changing it from 4 to 6.5 mm while the other parameters are kept constant at their initial values shown in Table 1. As the ${R}_{o}$ increases, the area enclosed by the ring increases. This leads to increasing the stored magnetic energy and hence increases the inductance. Therefore, the resonance frequency will be decreased as evident from Fig. 8 and Eq. 10. It is also evident from Fig. 8 that the maximum frequency shift with absorption above 80% occurs when ${R}_{o}$ equals to 4.5 mm when the refractive index of the analyte (n_a_) is changed from 1.3 to 1.35. Therefore, ${R}_{o}$ = 4.5 mm will be used for the next simulations. Table 2 summarizes the absorption peak, resonance frequency, and sensitivity over the studied range for different geometrical parameters, while only three representative values of each parameter are plotted in the figures for clarity.

**Table 2:** Absorption peak, resonance frequencies, and sensitivity at different values of the geometric parameters.

R_o_ (mm)	n = 1.3		n = 1.35		Sensitivity GHz/RIU
	Absorp	f_0_ (GHz)	A	f_0_ (GHz)	
4	0.98832	25.208	0.98500	24.868	6798.6
4.5	0.84505	23.78	0.81199	23.372	8160
5.5	0.96307	22.998	0.99579	22.828	3405.2
6	0.98190	22.352	0.97550	22.2017	3016.8
6.5	0.83058	22.284	0.78335	22.148	2720
**L**_**v**_** (mm)**					
1	0.627484	25.616	0.63915	25.344	5440
1.25	0.965237	24.766	0.96324	24.46	5782.4
1.75	0.852281	23.848	0.83450	23.474	7483.2
2	0.844672	23.78	0.81147	23.372	8175.6
2.25	0.852160	23.746	0.81373	23.304	8840
**D**_**o**_** (mm)**					
0.25	0.852158	23.746	0.81374	23.304	8840
1.25	0.998499	23.27	0.93900	23.032	4760
1.5	0.942187	22.827	0.98451	22.6916	2719
1.5	0.942187	22.827	0.98451	22.6916	2719
**D**_**i**_** (mm)**					
0.25	0.8522	23.746	0.81373	23.30	8840
0.5	0.8182	23.712	0.78102	23.27	8840
0.75	0.7902	23.712	0.75659	23.27	8840
1	0.8010	23.678	0.75091	23.24	8840
**W**_**h**_** (mm)**					
0.5	0.94152	23.542	0.926952	23.1	8840
0.75	0.92765	23.576	0.904812	23.134	9520
1	0.92186	23.576	0.896704	23.134	8840
1.25	0.90129	23.61	0.864171	23.168	9520
1.5	0.87161	23.644	0.835489	23.1	9520
**W**_**v**_** (mm)**					
0.25	0.85214	23.746	0.81373	23.304	8840
1	0.77143	23.78	0.73076	23.372	8160
1.25	0.75027	23.78	0.70716	23.406	7480
1.5	0.71993	23.848	0.68564	23.44	8160

**Fig. 9:**
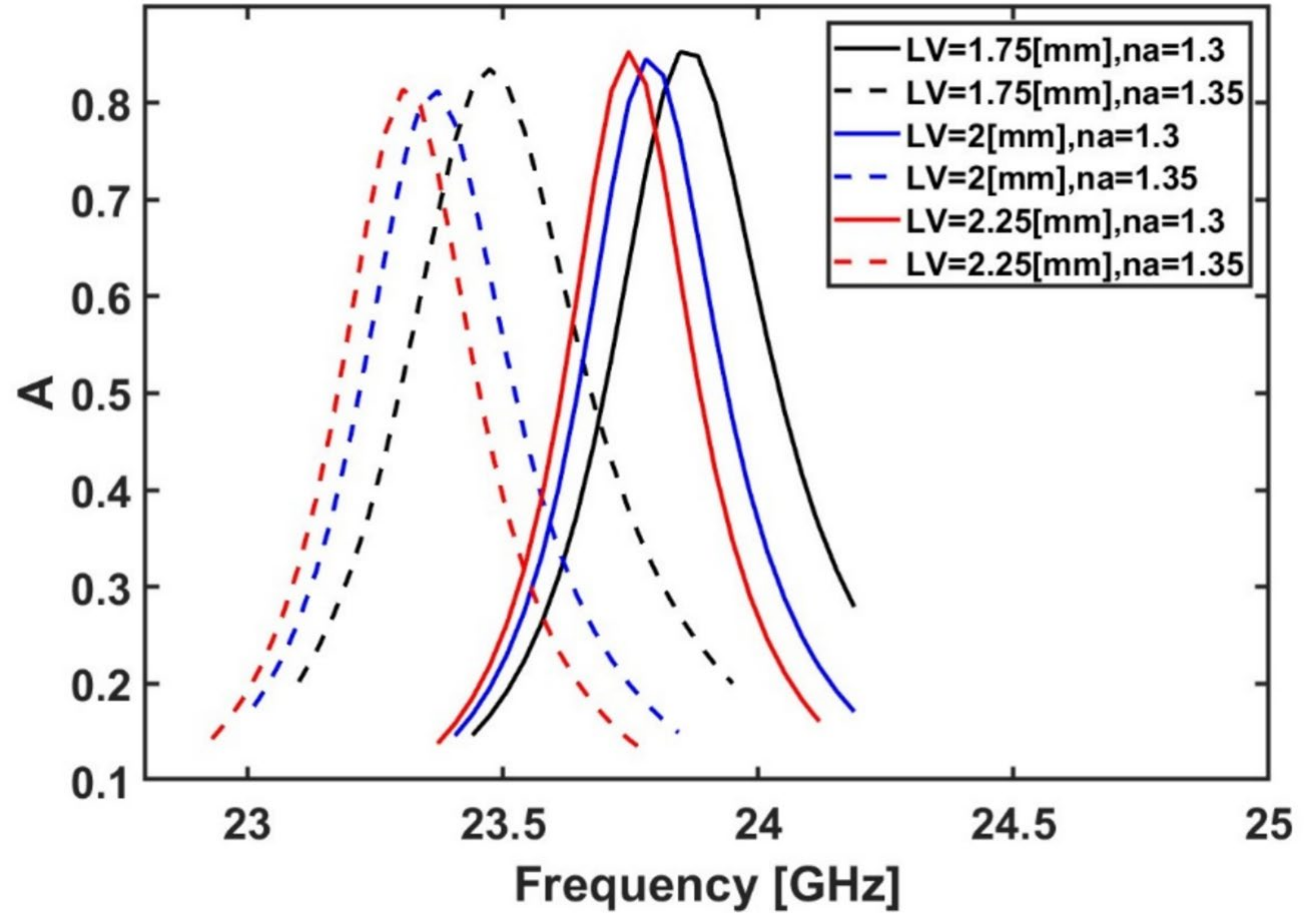
Variation of the absorption spectrum at different ${{\boldsymbol{L}}}_{{\boldsymbol{v}}}$ values while ${{\boldsymbol{R}}}_{{\boldsymbol{o}}}$ = 4.5 mm and the rest of the geometrical parameters remain constant at their initial values (${{\boldsymbol{R}}}_{{\boldsymbol{i}}}$ = 1.8 mm, ${{\boldsymbol{D}}}_{{\boldsymbol{i}}}$ = 0.25 mm, ${{\boldsymbol{L}}}_{{\boldsymbol{h}}}$ = 0.8 mm, ${{\boldsymbol{W}}}_{{\boldsymbol{h}}}$ = 0.25 mm, ${{\boldsymbol{D}}}_{{\boldsymbol{o}}}$ = 0.25 mm, and ${{\boldsymbol{W}}}_{{\boldsymbol{v}}}$ = 0.25 mm.)

The effect of the vertical open-circuit stub length ${L}_{v}$ on the sensor sensitivity is next studied by changing it from 1.0 to 2.25 mm. Figure 9 shows the impact of the variation of ${L}_{v}$ on the resonance frequency and the absorption. It is evident from Fig. 9 that the maximum frequency shift with absorptivity above 80% occurs when ${L}_{v}$ equals to 2.25 mm when the analyte’s refractive index is changed from 1.3 to 1.35. Consequently, the optimum value for ${L}_{v}$ is chosen to be 2.25 mm. It is worth noting that the open-circuit stub is modeled as a parallel capacitor when the length is smaller than a quarter wavelength^68^. Further, the length of the stub is directly proportional to the modeled capacitor. Consequently, by increasing ${L}_{v}$ the capacitance will be increased, which leads to a reduction in the resonance frequency as shown in Fig. 9.

**Fig. 10:**
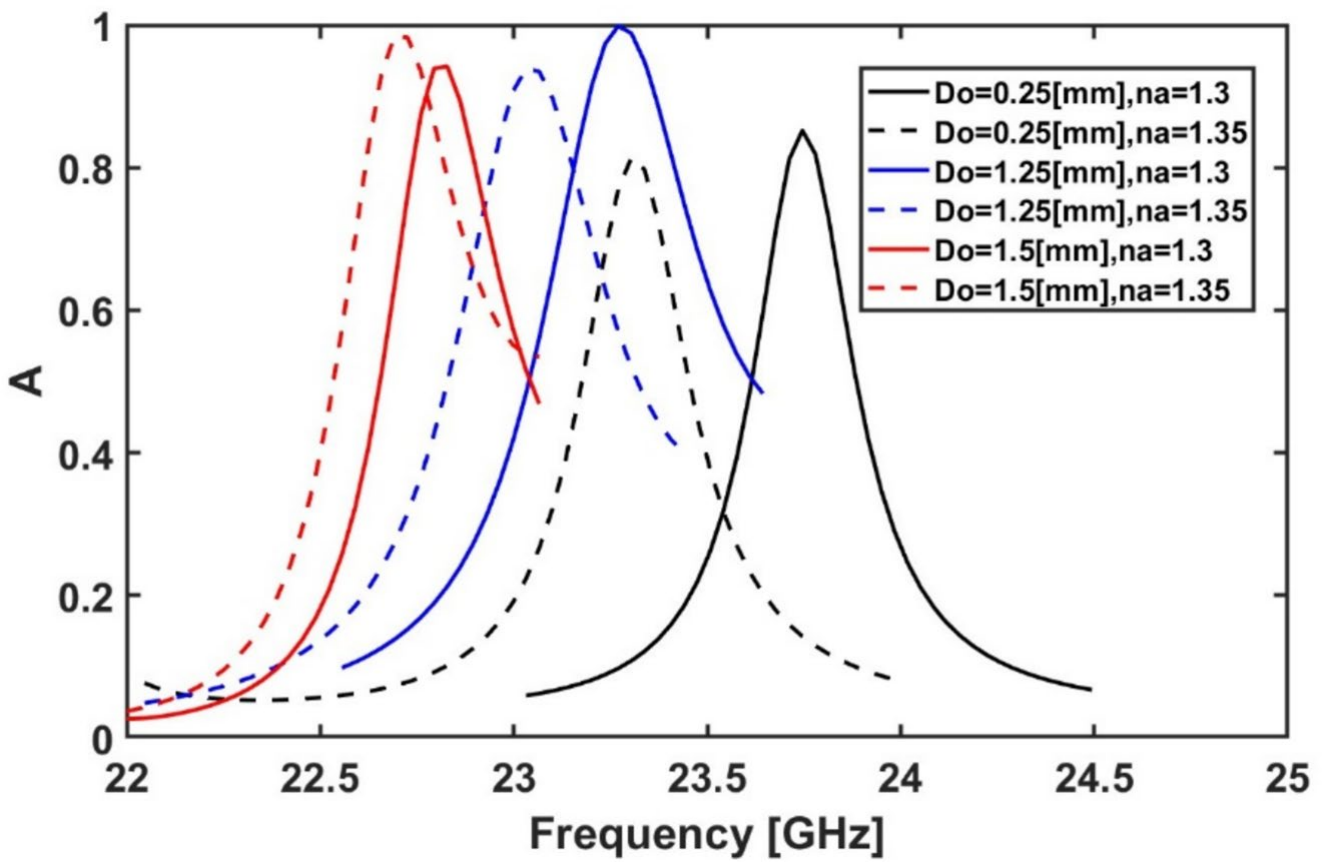
Variation of the absorption spectrum at different ${D}_{o}$ values while ${R}_{o}$ = 4.5 mm, ${L}_{v}$ = 2.25 mm and the rest of the geometrical parameters remain constant at their initial values (${R}_{i}$ = 1.8 mm, ${D}_{i}$ = 0.25 mm, ${L}_{h}$ = 0.8 mm, ${W}_{h}$ = 0.25 mm, and ${W}_{v}$ = 0.25 mm.)

Then, the thickness of the outer ring ${D}_{o}$ is studied by changing its value from 0.25 to 1.5 mm. As shown in Fig. 10, the maximum frequency shift with absorption above 80% occurs when ${D}_{o}$ equals to 0.25 mm when the refractive index of the analyte is changed from 1.3 to 1.35. Consequently, the optimum value for ${D}_{o}$ is equal to $0.25\text{ mm}$ for the subsequent simulations. It should be noted that the thickness of the outer ring affects both inductance and capacitance simultaneously. When the ring thickness increases, the current spreads out, which reduces the magnetic field density. As a result, the magnetic energy stored in the structure decreases, leading to a reduction in inductance. Since the inductance decreases, the resonance frequency tends to increase. However, increasing the ring thickness also increases the surface area available for storing charge. This leads to stronger electric field confinement and, consequently, an increase in capacitance. As the capacitance increases, the resonance frequency decreases. Therefore, increasing the ring thickness has two opposing effects. But a key factor to consider is that the inductance decreases gradually, while the capacitance increases more significantly. As a result, the overall effect is a decrease in the resonance frequency^68^.

**Fig. 11:**
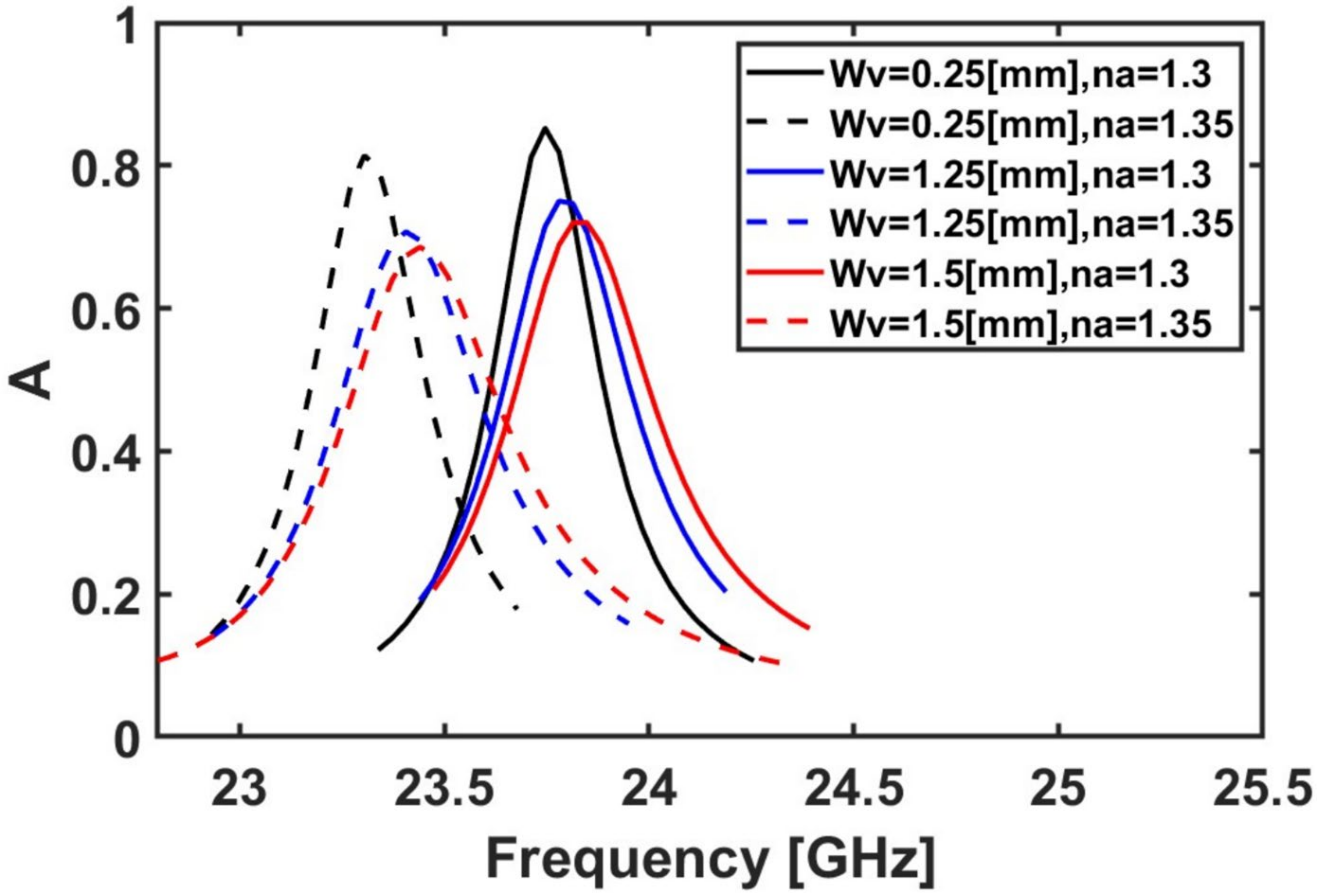
Variation of the absorption spectrum at different ${{\boldsymbol{W}}}_{{\boldsymbol{v}}}$ values while ${R}_{o}$ = 4.5 mm, for ${L}_{v}$ = 2.25 mm, for ${D}_{o}$ = 0.25 mm and the rest of the geometrical parameters remain constant at their initial values (${R}_{i}$ = 1.8 mm, ${D}_{i}$ = 0.25 mm, ${L}_{h}$ = 0.8 mm, and ${W}_{h}$ = 0.25 mm).

The impact of the vertical open-circuit stub ${W}_{v}$ thickness on the sensor sensitivity is also studied by changing its value from 0.25 to 1.5 mm. As shown in Fig. 11, the maximum frequency shift with absorption above 80% occurs when ${W}_{v}$ equals to 0.25 mm as n_a_ is changed from 1.3 to 1.35. Consequently, ${W}_{v}$ = 0.25 mm is used for the subsequent simulations. It is worth noting that the thickness of the open-circuit stub in our case affects both the model capacitance of the stub itself and the capacitance between the stub and the inner ring. As the thickness of the stub increases, the surface area also increases, leading to an increase in both types of capacitances. However, since these capacitances are effective in series, the overall equivalent capacitance decreases, and therefore, the resonance frequency increases.

**Fig. 12:**
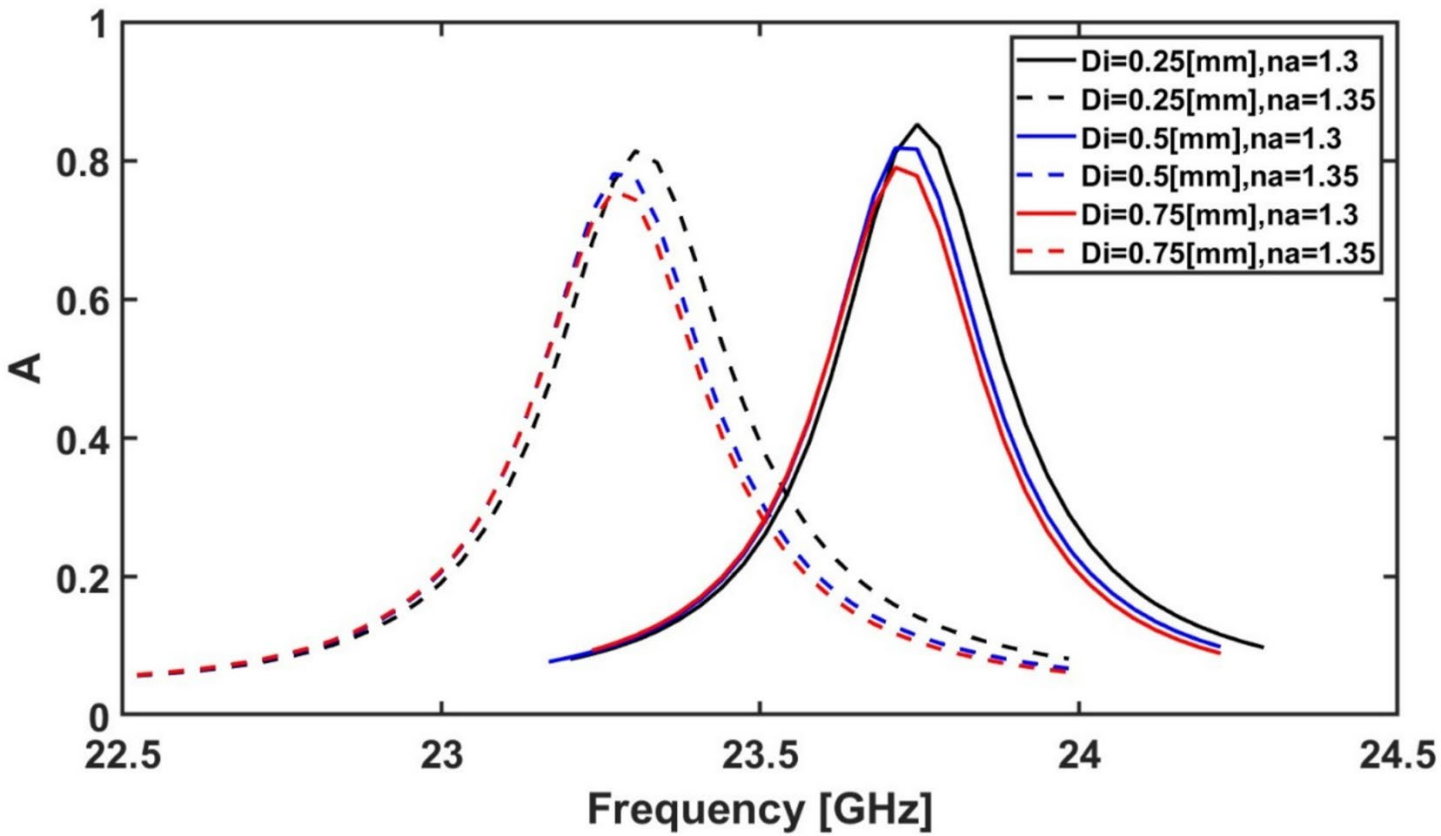
Variation of the absorption spectrum at different D_i_ values, while ${R}_{o}$ = 4.5 mm, for ${L}_{v}$ = 2.25 mm, ${D}_{o}$ = 0.25 mm, ${W}_{v}$ = 0.25 mm and the rest of the geometrical parameters remain constant at their initial values (${R}_{i}$ = 1.8 mm, ${D}_{i}$ = 0.25 mm, and ${L}_{h}$ = 0.8 mm).

The next studied geometrical parameter is ${D}_{i}$ by changing it from 0.25 to 1.5 mm. Figure 12 shows the effect of the variation of ${D}_{i}$ on the resonance frequency and the absorption. It is revealed from Fig. 12 that the maximum frequency shift with absorptivity above 80% occurs when ${D}_{i}$ equals to 0.25 mm when n_a_ is changed from 1.3 to 1.35. Consequently, ${D}_{i}$ = 0.25 mm is selected as an optimum value for the proposed sensor. It should be noted that the thickness of the outer ring impacts both inductance and capacitance simultaneously. When the ring thickness increases, the current spreads out, which reduces the magnetic field density. As a result, the magnetic energy stored in the structure decreases, leading to a reduction in the equivalent inductance. Since the inductance decreases, the resonance frequency tends to increase. However, increasing the ring thickness also increases the surface area available for charge storing. This leads to stronger electric field confinement and, consequently, an increase in the equivalent capacitance. As the capacitance increases, the resonance frequency decreases. Therefore, increasing the ring thickness has two opposing effects. But a key factor to consider is that the inductance decreases gradually, while the capacitance increases more significantly. As a result, the overall effect is a decrease in the resonance frequency^68^.

**Fig. 13:**
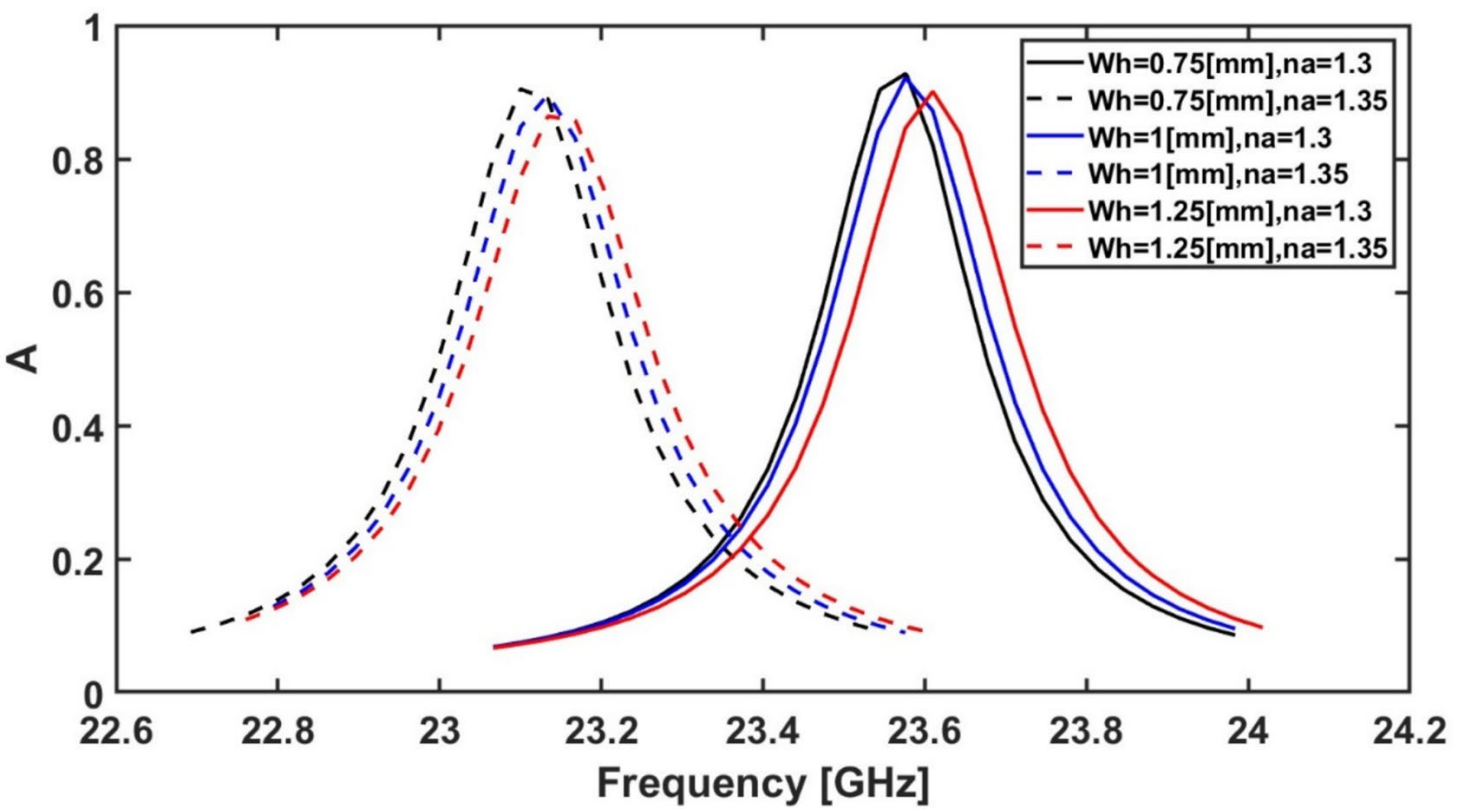
Variation of the absorption spectrum at different ${W}_{h}$ values while ${R}_{o}$ = 4.5 mm, ${L}_{v}$ = 2.25 mm, ${D}_{o}$ = 0.25 mm, ${W}_{v}$ = 0.25 mm, ${D}_{i}$ = 0.25 mm and the rest of the geometrical parameters remain constant at their initial values (${R}_{i}$ = 1.8 mm, and ${L}_{h}$ = 0.8 mm).

Additionally, ${W}_{h}$ is next studied by changing its value from 0.25 to 1.5 mm. Figure 13 shows the effect of the variation of ${W}_{h}$ on the absorption response of the suggested MMA. It may be seen from Fig. 13 that the highest frequency shift with absorption above 80% is achieved at ${W}_{h}$ equal to 0.75 mm as the analyte refractive index is changed from 1.3 to 1.35. Consequently, the optimum value for ${W}_{h}$ is equal to 0.75 mm.

Finally, the impact of the ${L}_{h}$ is investigated by changing its value from 0.8 to 1.8 mm. It is found that the absorption response is almost constant with the variation of ${L}_{h}$ which results in no change in the sensor sensitivity. The optimum geometrical parameters are summarized in Table 3.

**Table 3:** The optimum geometrical parameters of the proposed MMA sensor.

Parameter	R_o_	R_i_	L_v_	L_h_	D_o_	D_i_	W_v_	W_h_
Value (mm)	4.5	1.8	2.25	0.8	0.25	0.25	0.25	0.75

**Fig. 14:**
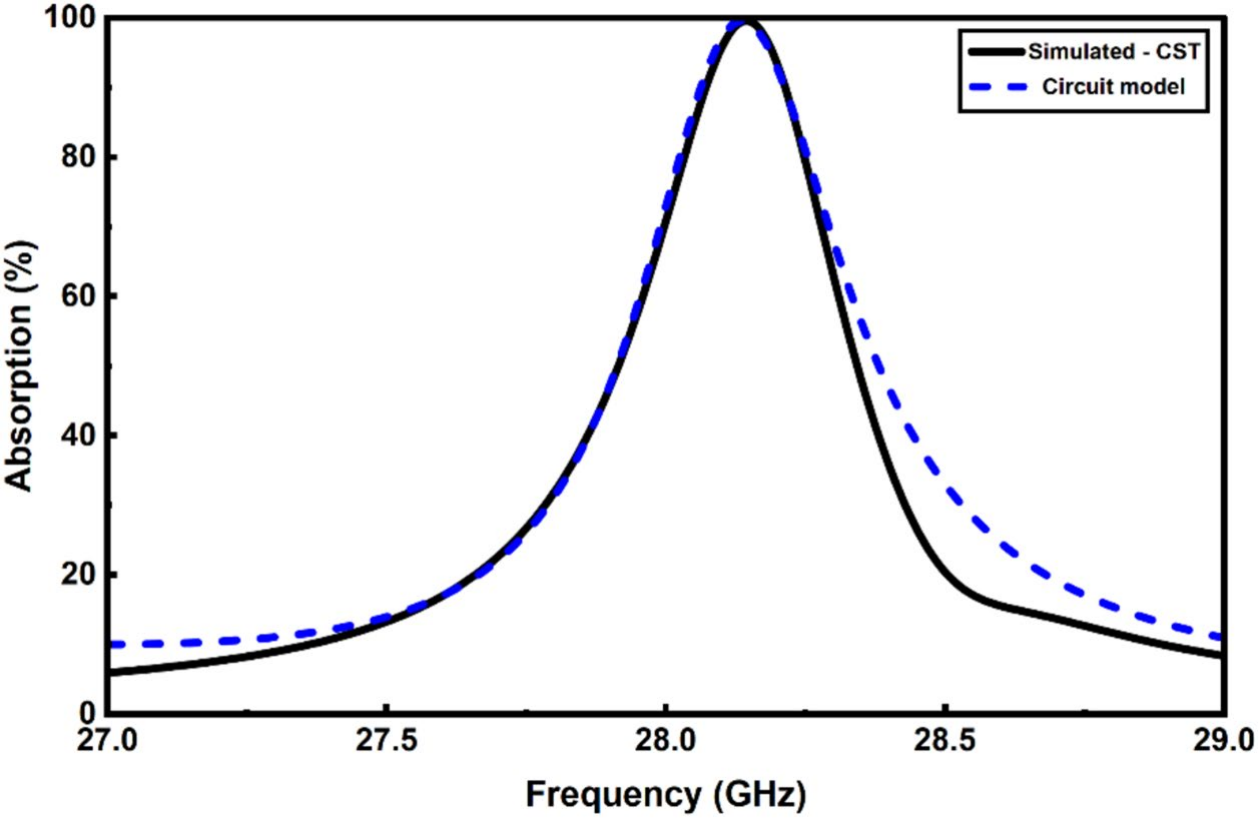
Comparison between the calculated absorption of the MMA by using the CST and the circuit model.

The circuit parameters shown in Fig. 6 are then extracted using the optimum geometrical parameters summarized in Table 3. In this context, it is found that R_1_ = 40 Ω, L_1_ = 32.75 nH, C_1_ = 1 fF, L_d_ = 50 nH, R_d_ = 88 Ω, Z_1_ = 160 Ω (with an electrical length 82°) and Z_2_ = 25 Ω (with an electrical length 81°). As shown in Fig. 14, the absorption curve obtained by the circuit model aligns well with the full-wave simulation results from CST. This good agreement confirms that our circuit model is a highly reliable tool for characterizing the MMA’s behavior.

Table 4 shows the performance of the proposed metamaterial refractive index sensor relative to those reported in the literature. To compare the sensitivity of sensors that operate in different frequency ranges, the normalized sensitivity will be used which can be calculated from the following equation^62^

**Table 4:** The performance of the reported metamaterial refractive index sensor relative to those reported in the literature.

References	Dimensions of unit cell (mm)	Frequency Range (GHz)	Absorptivity (%)	Resonance Frequency (GHz)	ΔF (MHz)	Normalized Sensitivity S (%) ${\mathrm{RIU}}^{-1}$	Q-Factor	FoM	Application
^29^ Jul. 2022	22.86 × 10.16	8–12	96.83	9.06	110	3.088	435	13.432	Detection of materials and their thickness
			88.38	11.16	150	2.803	325	9.109	
^27^ Dec. 2022	22.86 × 10.16	8–12	99.5	9.46	70	1.409	135	1.902	Liquid sensing
^31^ Feb. 2023	8 × 8	4–20	–	6.24	–	–	62.4	–	–
			–	10.608			17.68		
^28^ Mar. 2023	22.86 × 10.16	8–12	99.99	10.21	410	5.45	430	23.435	Fuel and oil adulteration sensing
^30^ Dec. 2023	20 × 20	2–4	99.9	2.88	62.821	2.032	1413.29	28.718	Material characterization and sensing
			98.9	3.57	22.384	0.584	1140.16	6.658	
^71^ Dec. 2023	15 × 15	2–14	99.92	5.46	920	15.56	665.85	103.606	–
			99.98	11.3	1660	13.1	1883.3	246.712	
^32^ 2024	10 × 10	18–34	99.9	20.193	–	–	155.33	–	–
			99.9	30.087			527.84		
This work	15 × 15	20–30		28.146	4650	51.623	817	427	Detection of Cancer Cells

11 $$Normalized Sensitivity= \frac{Sensitivity(S)}{Resonance frequency({f}_{o})} {RIU}^{-1}$$

It is evident from Table 4 that the proposed MMA sensor offers several advantages, including a compact size, higher sensitivity compared with previously reported works, and a large Q-factor. Although Refs.^30^ and^32^ exhibit higher Q-factors, their figures of merit (FoM) are lower than that of the proposed sensor, whose FoM significantly surpasses those reported in the literature.

**Fig. 15:**
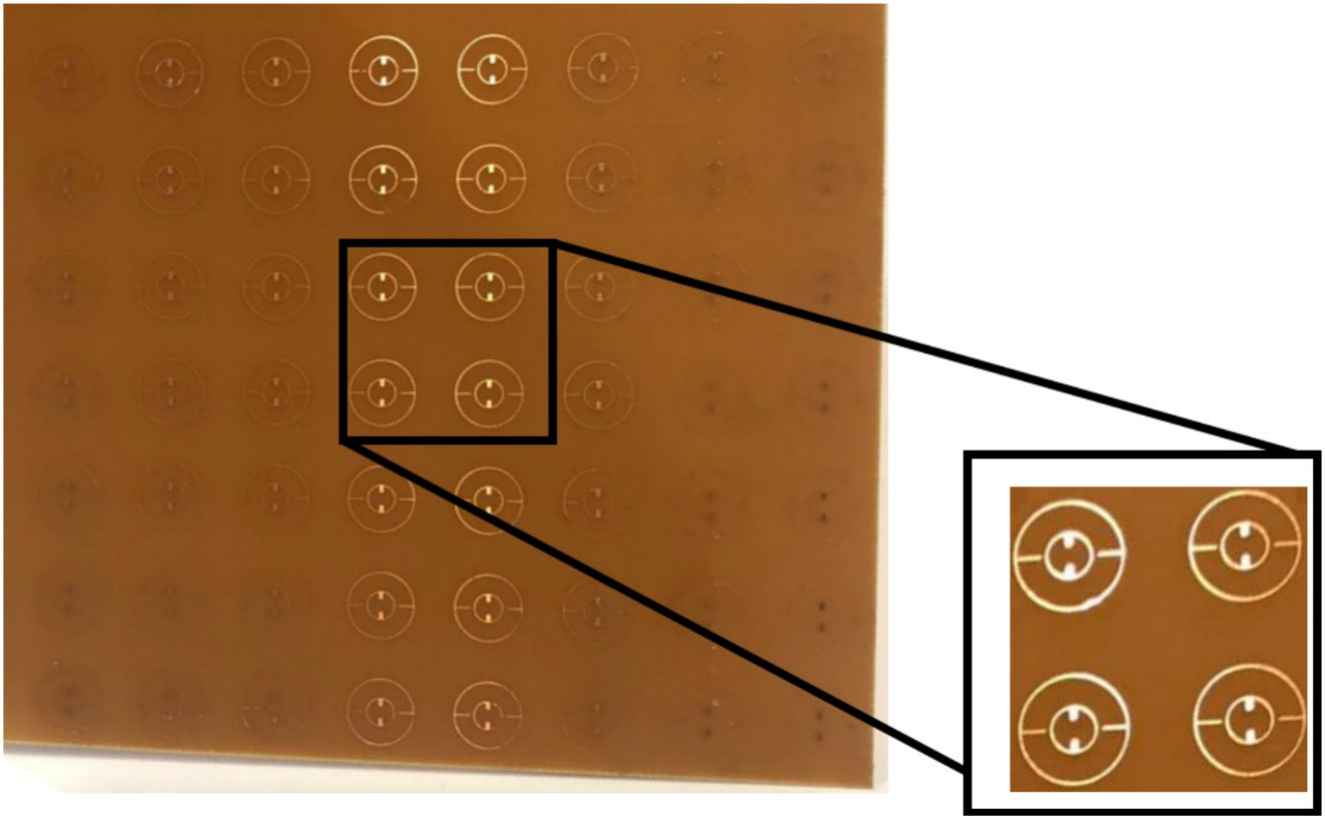
Prototype of the proposed perfect MMA.

To experimentally validate the effectiveness of the proposed MMA sensor, a prototype was created using the optimal geometric parameters through the photolithography technique as shown in Fig. 15. The completed prototype measures 150 × 150 × 0.8 mm and consists of 10 × 10-unit cells. It is fabricated on an FR-4 substrate coated with copper on both sides, featuring a copper layer thickness of 0.035 mm.

**Fig. 16:**
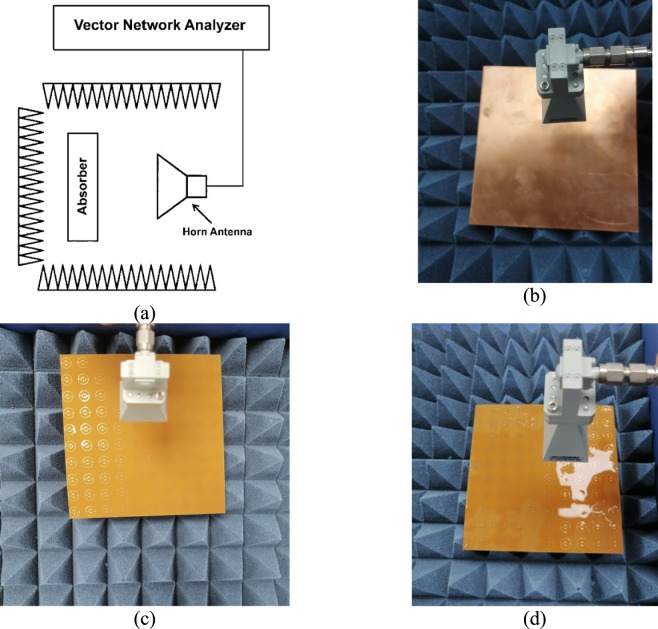
(**a**) A schematic representation of the measurement setup for the (**b**) perfect reflector with the same dimensions (used as a reference), (**c**) the suggested MMA with no analyte layer, (**d**) the suggested MMA with water layer.

**Fig. 17:**
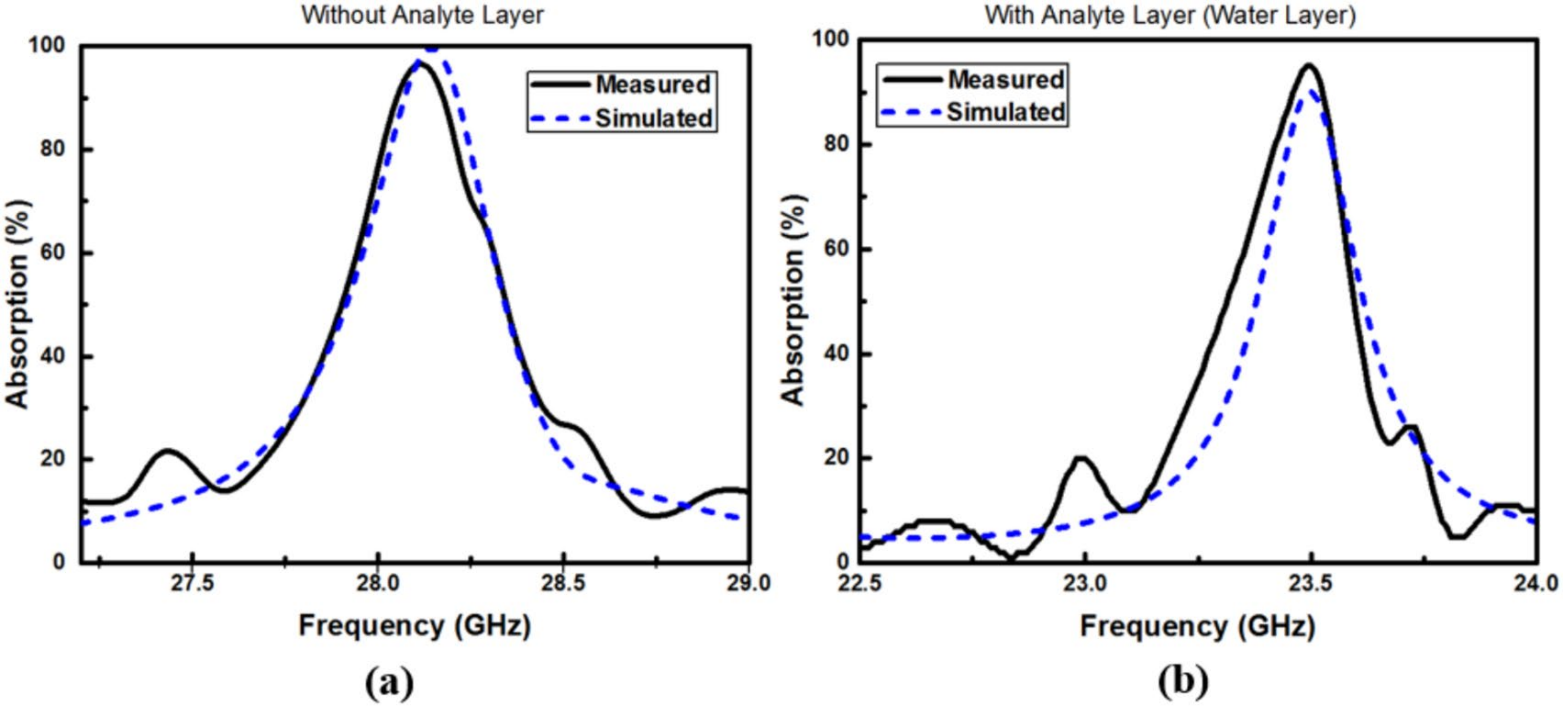
Measured and simulated absorption spectrum (**a**) without and (**b**) with an analyte (water) layer.

To measure the S-parameters of the suggested MMA and hence determine the absorption behavior, a measurement setup shown in Fig. 16a is used. In this context, the S-parameters can be measured using a horn antenna, along with a vector network analyzer (AgilentFieldFox® model N9918A). Initially, the S-parameters of a perfect reflector are measured as a reference object (Fig. 16 b). Then, the S-parameters of the proposed MMA are measured without (Fig. 16c) and with an analyte layer (Fig. 16d). The measured and simulated absorption of the reported MMA without/with an analyte layer are represented in Fig. 17a and b, respectively. It may be seen from this figure that there is a slight difference between the simulated and measured results. This difference arises from the influence of diffraction and scattered waves^70^, as the fabricated sample does not fulfill the ideal infinite geometry conditions. As a result, edge diffraction may take place during the measurement process. Additionally, minor deviations could occur during the fabrication stage.

Many real-world samples exhibit refractive indices within the range of 1.3–1.39. For example, the refractive index of healthy human blood is 1.35, while blood infected with T-type leukemia (Jurkat) has a refractive index of 1.39^72^. Additionally, normal basal cells (at a concentration of 40–60%) have n = 1.36, whereas cancerous basal cells (at 80% concentration) show a refractive index of 1.38^40^. These observations highlight that most biomedical samples fall within the refractive index range of 1.3–1.39. Consequently, the sensor under discussion demonstrates high accuracy for biomedical applications.

**Fig. 18:**
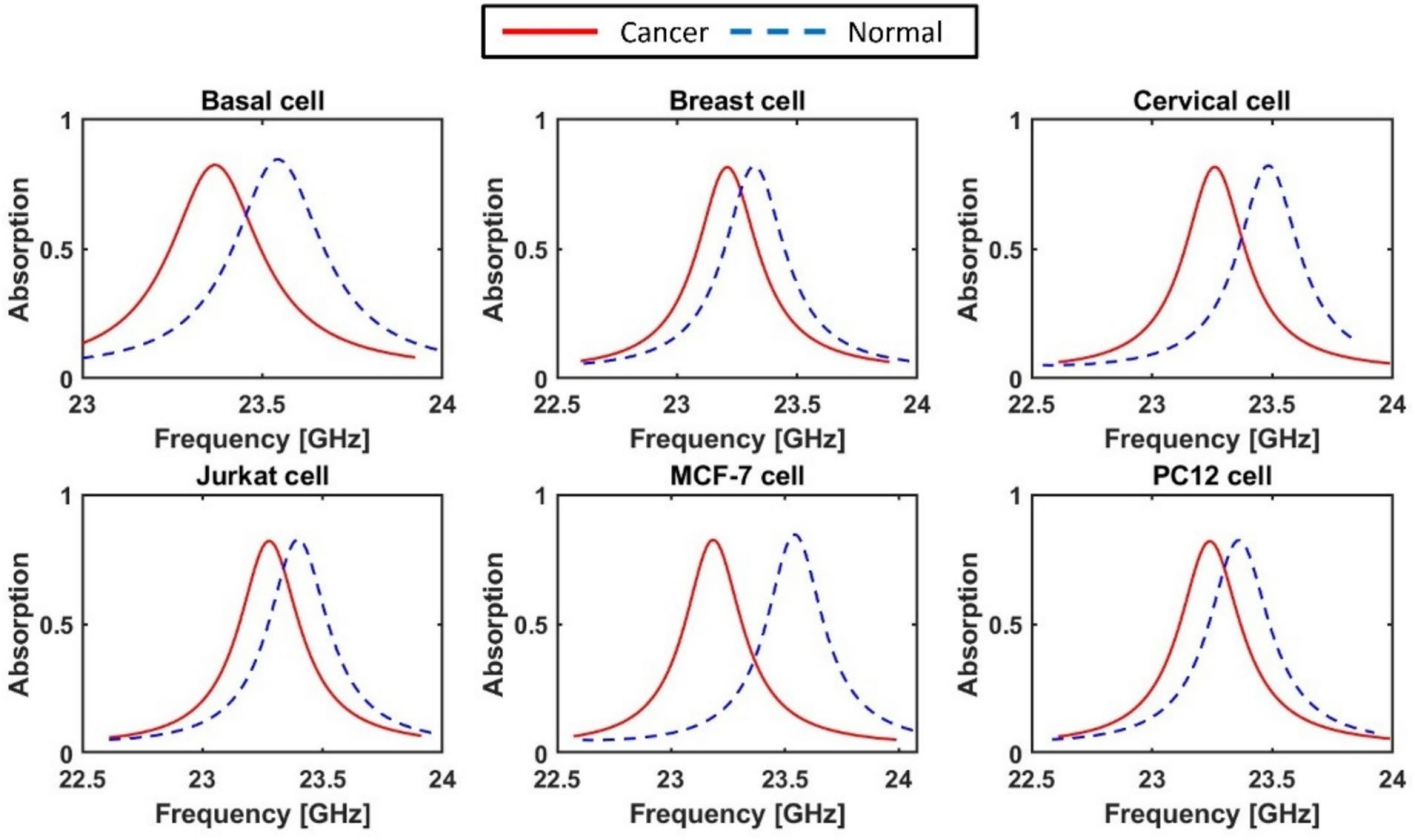
Variation of the absorption spectrum of the proposed sensor for various cancer cell types and their respective normal cells; (**a**) Basal, (**b**) Breast, (**c**) Cervical, (**d**) Jurkat, (**e**) MCF-7, and (**f**) PC12.

Cancer cells contain higher levels of protein with a higher refractive index compared to normal living cells^40^. Therefore, it becomes feasible to distinguish normal cells from cancerous ones using a refractive index sensor. The refractive indices of normal and cancerous cells are summarized in Table 5^40^. Hence, the proposed metamaterial biosensor can be used to facilitate early cancer detection in different organs based on their unique resonance frequency signatures. Figure 18 illustrates the relationship between the absorption coefficient and resonance frequency for various cancer cell types alongside their normal counterparts. The summarized results are presented in Table 5. From Fig. 18a, it is observed that basal cancer cells exhibit a resonance frequency of 23.365 GHz, while normal cells show a resonance frequency at 23.547 GHz. Such an expected increase in the resonance frequency is due to the reduction in the refractive index that leads to a decrease in the effective capacitance, thereby shifting the resonance toward higher frequencies. The resulting frequency shift is 0.182 GHz, corresponding to a sensitivity of 9.1 GHz/RIU. Similarly, breast cancer cells and normal cells resonate at 23.209 GHz and 23.326 GHz, respectively, with a sensitivity of 8.36 GHz/RIU, as shown in Fig. 18b and Table 5. Additionally, the analysis in Table 5 and Fig. 18c and d reveal sensitivities of 9.21 GHz/RIU and 9.29 GHz/RIU for cervical and Jurkat cancer types, corresponding to frequency shifts of 0.221 GHz and 0.13 GHz, respectively. For the MCF-7 cancer type, a frequency shift of 364 GHz and a sensitivity of 8.89 GHz/RIU are determined, as depicted in Fig. 18e. Furthermore, Table 5 and Fig. 18f indicate that the PC12 cancer cells have resonance frequencies of 23.235 GHz and 23.365 GHz, achieving a sensitivity of 9.29 GHz/RIU.

**Table 5:** The sensitivity of the proposed sensor for different cancer cells^40^.

Cell Type	State (refractive index)	Resonance frequency (GHz)	Normalized Sensitivity S (%)${\mathrm{RIU}}^{-1}$	Sensitivity (GHz/RIU)
Basal cell	Cancer (1.38)	23.365	38.947	9.1
	Normal (1.36)	23.547		
Breast cell	Cancer (1.399)	23.209	36.008	8.36
	Normal (1.385)	23.326		
Cervical cell (Hela)	Cancer (1.392)	23.261	39.587	9.21
	Normal (1.368)	23.482		
Jurkat cell	Cancer (1.39)	23.274	39.897	9.29
	Normal (1.376)	23.404		
MCF-7 cell	Cancer (1.401)	23.183	38.295	8.89
	Normal (1.36)	23.547		
PC12 cell	Cancer (1.395)	23.235	39.964	9.29
	Normal (1.381)	23.365

The performance of the proposed MMA sensor is evaluated in comparison with previously reported MMA sensors. Table 6 presents a comparative analysis, highlighting key parameters such as operating frequency range, normalized sensitivity, Q-factor, and FoM. From Table 6, it is evident that the proposed MMA sensor offers high performance with higher sensitivity than others in the literature, except for^58^ and^59^, which have larger sensitivity. However, the proposed work has a Q-factor and FoM that surpass those reported in the literature by a large margin.

**Table 6:** The performance of the reported structure as a biosensor relative to those reported in the literature.

References	Resonance Frequency	Absorptivity (%)	Normalized Sensitivity S (%)${\mathrm{RIU}}^{-1}$	Q-Factor	FoM ${\mathrm{RIU}}^{-1}$	Bio-application
^62^ 2022	105.7 GHz	99.7	0.14	19.57	3.48	Refractive index detection of biomedical samples
^61^ 2023	2.988 GHz	94.1	0.061	–	–	Skin, blood, and Breast cancer
^41^ Dec. 2023	4.101 THz	94.313	49.987	55.34	25.24	Skin, blood, and Breast cancer
^60^ Mar. 2024	36.508 GHz	99.9	0.17	23.1	1.554	Skin, blood, Cervical, and Breast cancer
^59^	0.4728 THz	99.8	196.7	24.86	42.6	Blood cancer
	0.69826 THz	99.9	233.437	24.83	50.6	
	0.7901 THz	99.9	321.478	34.74	98.6	
^58^ Aug. 2024	5.662 THz	99.1	68.52702	8.948	8.146	Skin, blood, cervical, adrenal gland, and breast cancer
^56^ Feb. 2025	0.684THz	–	7.163743	85.77	6.3	Cervical cancer
	0.971 THz	–	7.00309	41.46	3	
^57^ 2025	3.257 THz	98.84	27.54068	325.7	89.7	Cutaneous malignancies
This work	28.146 GHz	99.33	51.62368	817	427	Skin, blood, Cervical, and Breast cancer

## Conclusion

Efficient MMA is reported, fabricated and characterized for sensing applications. Initially, the finite integral method via CST simulation program is used to study the suggested metamaterial absorber. The proposed design has a simulated absorptivity of 99.33% at 28.146 GHz, which falls within the mm-Wave spectrum, a band of critical importance for next-generation 5G communication and high-frequency biosensing. Further, the reported absorber has a simulated high Quality factor Q of 817 and FoM of 427. A prototype of the suggested absorber is fabricated and characterized, which shows a good agreement with the simulated results. The experimental results reveal that a high sensor sensitivity of 14.44 GHz/RIU is achieved with a Q-factor of 513. Then, the proposed MMA is introduced as a biosensor for early cancer detection for Breast cell, Cervical cell, Jurkat cell, MCF-7 cell, and PC12 cell with an average sensitivity of 9.023 GHz/RIU. Accordingly, the fabricated perfect metamaterial absorber could act as a promising candidate for sensing applications.

## Data Availability

All data supporting the findings of this study are available within the submitted paper
